# What Can Faculty Members and Programs Do to Improve Students' Learning?

**DOI:** 10.5402/2011/649431

**Published:** 2011-11-03

**Authors:** Alice P. Gaudine, Donna G. Moralejo

**Affiliations:** School of Nursing, Memorial University of Newfoundland, 300 Prince Philip Drive, HSC H2916, St. John's, NL, Canada A1B 3V6

## Abstract

*Purpose*. To understand what faculty members can do to improve students' learning, we explored (1) program and course characteristics important to students, (2) choices within courses students feel are important, and (3) students' perceptions of learning online. *Background*. Web delivery alters interactions among students and faculty, thereby changing the learning environment. *Methods*. Master of Nursing students currently enrolled in one graduate program were invited to participate in a web-based survey. *Findings*. The students valued (1) frequent feedback and interaction with the instructor, (2) organized, structured, and current content, (3) flexibility in deadlines and participation, (4) seamless navigation and technical support, and (5) choices in assignments. Students perceived their learning in online courses as less than if they had taken the course on campus. *Conclusions*. Professors can address some of the items identified by students as important, such as being flexible with due dates and expectations for participation in online discussion.

## 1. Introduction

Distance education has increased access to both undergraduate and graduate education for nurses. The characteristics of distance education that promote accessibility and independent study, such as interaction through web-based media, also change the learning environment. Student-student and student-professor interactions change, especially when much of the interaction is asynchronous and text based. While there is potential to get to know students individually, spontaneity of discussion can be inhibited. Depth of discussion may also be reduced as slow typists may find it frustrating to elaborate on ideas. Videoconferencing, to promote synchronous discussion, is not always an option, depending on the availability of technology and hampered by time zone differences.

Numerous studies have identified issues that both threaten and facilitate learning by distance education [1]. From these, strategies have been recommended to promote learning. For example, technical difficulties or lack of computer skills lead to students' frustration, anxiety, and confusion, which could both interfere with learning and lead to student dissatisfaction [1]. Designing course web sites that are easily navigated, providing orientation to the software, and providing accessible technical support for prolonged hours are examples of strategies that address this particular issue. Some issues are more complex, such as facilitating collaborative learning, and thus require multiple strategies [2].

As online teaching strategies have been developed, more attention has been paid to evaluating their effectiveness. Blood-Siegfried et al. [3] have compiled a helpful rubric for evaluation of Master of Nursing (MN) courses. Their criteria are based on the best recommendations for strategies that promote learning, with five categories: organization and design, course content, instruction, interaction, and evaluation and assessment. What has not been done, however, is distinguishing between those elements that are controllable by individual professors, such as content, and those that must be addressed at a program or institution level, such as choice of web platform.

One such element that requires further study is that of learner choice, which is related to, but not identical to, flexibility. The literature on learner choice is sparse but generally related to choice of electives in a curriculum or sequencing of courses. Flexibility has been related to improving access to education since students do not need to move to a new area and can continue to work. School work can be done from home, at hours convenient to the individual [4]. For a number of years, flexibility has been discussed in the context of assessing individual learning styles, providing different instructional approaches so that students can access resources to match their own learning styles, and individualizing study plans within a program [5]. The actual impact of adapting instructional approaches to learning styles has not been clearly demonstrated [6]. Little has been written on what elements in a course or program students would like to be able to make choices about.

Multiple studies have compared learning outcomes by distance and traditional classroom education. Many studies have methodological weaknesses, such as inadequate control groups or small sample sizes. In general, however, no major differences have been found between distance and traditional education approaches in terms of changes in knowledge or student satisfaction [7, 8]. Maring et al. [8], for example, taught pathophysiology to the same group of 96 physiotherapy students via traditional classroom and web-based methods, with the students instructed by one method one term and by the other method the following term. The mean exam mark for the distance course, 83.2%, was slightly better than the mean exam more for the classroom course, 78.9%. Comments from the students, however, suggested that they thought they learned better in the classroom setting.

While many studies have assessed student satisfaction with online learning, few have looked at student perceptions of learning by distance education compared to classroom learning. Maring et al. [8] did so indirectly, by looking at students' general comments, while Mitchell et al. [9] did so directly. They asked 27 dental hygiene students, who had taken at least one distance education course in their program, whether they expected to learn the same amount of course content in the web-based class as they would if the course were offered in the traditional classroom setting. The average score for the item was 4.44, on a 5-point Likert-type scale, where 1 was strongly disagree and 5 was strongly agree. Similarly, they agreed, with a mean score of 4.46, that they expected to achieve the same grade in the online course as in a classroom room. Having similar expectations for learning is important if students in distance education programs are to believe they receive the same quality education as those who attend classes on-campus.

To further understand what individual faculty members can do to facilitate learning in a MN program, as well as to understand students' perception of their learning, we conducted a descriptive study using an online survey to answer the questions:

1. What program and course characteristics do MN students feel are important for their satisfaction with online learning?
2. What types of choices provided within courses do MN students feel are important to them?
3. What are MN students' perceptions of their learning?

## 2. Methodology

This descriptive study surveyed MN students at one university, using an online questionnaire.

### 2.1. Setting and Sample

The study was conducted at a university school of nursing in Atlantic Canada which, in September 2002, was the first Canadian university to admit students into a Master in Nursing program offered by web delivery. Students were eligible to participate if they were enrolled in the program at the time of the study and had completed at least one course via the online route.

### 2.2. Data Collection Method

In spring of 2005, an invitation to participate in the study was sent by email to all MN students enrolled in (MN) program at the time of the study, with a statement that only those students who had taken at least one online course during their program should complete the questionnaire. The email included a link to the online survey questionnaire and explained that the purpose of the survey was to evaluate their responses to web delivery of graduate courses. They were informed that the survey questionnaire would take about 15 minutes to complete, their responses would be anonymous, and no one at the university would be able to link their name with responses, or even know if they responded. A second email was sent approximately 2 weeks later to remind students to complete the online questionnaire.

Consent to participate in the study was implied by the graduate student completing the online survey questionnaire. Ethical approval for the study was obtained from the research ethics committee at the university where this study was conducted.

### 2.3. Measures

The online survey questionnaire was developed for this study. It consisted of three sets of items related to their ideal program, choice and learning. Each item used a 5-point Likert-type scale; the graduate students were asked to rate their responses to each item on a scale from (1) strongly disagree to (5) strongly agree. This study was actually part of a larger program evaluation, with additional items addressing their actual experience with program; the program evaluation results will not be discussed in this paper. 

#### 2.3.1. Ideal Program

Students' perception of their ideal program was measured with 20 items covering a variety of topics related to the program as a whole and to courses within an ideal program. Sample items are “my ideal program would provide opportunities for me to respond to the ideas of other course participants,” “my ideal program would encourage team effort and community building among learners,” “my ideal program would have courses that have frequent discussion forums,” and “my ideal program would have instructors that respond to my emails within 24 hours.”

#### 2.3.2. Preference for Choice and Flexibility

Students' preferences for choice and flexibility in their courses and program was measured with 12 items (Cronbach's alpha =  .77). Sample items are “in my ideal program, all courses provide for choice in assignment topics,” “I would prefer that all courses provided choice in terms of how many discussion forums I was required to participate in,” and “I should be able to exercise some autonomy in terms of which weeks of the course I do more or less of the coursework.”

#### 2.3.3. Learning

Students' perception of their learning was measured with 7 items (Cronbach's alpha = 0.85). Sample items are “I feel that I can apply my new knowledge to new and different situations in my professional life,” “I am satisfied with my level of learning in most of my online courses to date,” and “I feel that my learning outcomes from online courses are as favorable as if I had taken the course on campus.”

In addition, the questionnaire included items about the students' backgrounds, questions inviting additional comments, and three additional questions. Two of these were “yes or no” questions that asked (1) if one or two brief courses (4 weeks) on campus would have improved learning; (2) if they would have registered for the program if one or two mandatory courses (4 weeks per course) had been included in the program. The third open-ended question asked students who felt a mandatory on-campus course would improve their learning to explain what they felt would be improved.

### 2.4. Data Analysis

Data were analyzed into SPSS 17.0 for Windows. Descriptive statistics were used to summarize the mean ratings given to each item. Content analysis was done on the written comments submitted, with themes identified.

## 3. Results

The invitation to participate was sent to the entire population of 93 MN students enrolled in the program; five students had not taken an online course. Of the 88 students eligible to participate in the study, 45 responded for a response rate of 51.1%. As shown in Table 1, most of the respondents were female, were older than age 40, worked more than 30 hours per week, and spent 2 to 5 hours per week on the discussion forum. 

Table 2 summarizes the mean scores of aspects related to an *ideal program*. Items which scored the highest were “my ideal program would provide for a leave of absence status without financial penalty,” “…would have technical support available within 24 hours,” and “would be supported by a strong administrative infrastructure.” Table 2 also summarizes the mean scores of aspects about *courses* within an ideal program. The highest scores were for items related to *content and structure*. Scores indicate that it was important to the students to have courses that are well organized, with seamless navigation, and that content is meaningful, current, and related to assignments. “Courses provide opportunities for me to receive feedback about my ideas from course instructors” had a higher mean score, 4.56, than the other items related to collaboration, feedback, and discussion forums, although all items about collaboration and feedback had a mean score of greater than 4. Response time of instructors to emails was less important than obtaining feedback: the item “instructors respond to my emails within 24 hours” had a mean score of 3.78.

Although students rated items related to collaboration and feedback fairly highly, the item “courses have frequent discussion forums” had one of the lowest mean scores (3.47). Comments suggested that online discussions were viewed as time consuming while adding little value by some students. One student wrote “I feel the online discussion forums to be limiting in my ability to share thoughts and feelings. In addition, the number of postings can be quite overwhelming and may have little added value when students are just posting for the sake of posting.” 

As shown in Table 3, in the items related to *choice*, choice in assignment topics had a higher mean score, 4.40, than other items related to *flexibility*, though students indicated they would like flexibility in deadlines and participation (mean scores: 3.91–4.02).

Table 3 also summarizes the scores related to *learning*. The item with the highest mean scores for students' perceptions of learning was “I feel I can apply my new knowledge to new and different situations in my professional life” (mean: 4.13). The item “I am satisfied with my level of learning in most of my online courses to date” had a mean score of 3.84, while the item that asked students if they felt their learning outcomes from online courses were as favorable as if they had taken the course on campus had a mean of 3.08.

 In response to the question “if your distance program had included one or two brief courses (4 weeks) at the university's campus, do you feel this would have improved your learning in the program?” 17 responded *no* and 12 responded *yes*. In response to the question “if the distance program had included one or two mandatory courses (4 weeks per course) at the university's campus, do you feel you would have registered for this program?” 20 responded *no* and 9 responded *yes*. 

Ten students responded to the open-ended question about what could be gained from a mandatory on-campus course. Their comments suggest they miss the discussions and support that are part of on-campus courses. One student commented “campus courses could also offer the opportunity to network and build lasting relationships with other students.” Another commented “many times during the distance program I have felt socially isolated, which has had a negative effect on my learning.”

The responses also suggested that actual learning may be improved with on-campus courses. “The face-to-face dialogue is always better than the written word of our online discussions and can provide timely clarification and exploration of the particular discussion.” Another student responded “…so much can be obtained from on-site delivery, body language and spontaneous discussion is lost on the web.”

## 4. Discussion and Implications

In this survey, many of the items with the highest scores related to issues best addressed by the school's administration: provision for a leave of absence without financial penalty, provision of technical support within 24 hours, and having a strong administrative infrastructure. Having seamless navigation in courses and receiving feedback from instructors, also amongst the highest scoring items, have implications for administration as well as for individual faculty members. The decision as to web platform to use is made by the university, for example, so it is important that their choice has sufficient features to promote easy navigation within courses. 

The MN students appreciated feedback from faculty members and their active participation in web discussions and would like to see more feedback and professors' participation in web discussions. Further, they would like a response to emails within a day. The amount of time it takes a faculty member to provide the level of support students require and/or anticipate in distances courses needs to be considered when making teaching assignments. Administrators therefore need to take into consideration not only faculty workload but student workload when determining the ideal number of students in a graduate web courses, as more participants may increase the number of postings and time of both faculty members and students. 

Strategies that support faculty feedback to graduate students and participation in web discussion may assist with student's learning. It is clear from the students' responses on this survey that they value interaction with faculty members through web discussion and emails. Alternatives to reduced class size may be strategies within a course that decrease the number of postings, such as limiting the number of postings per student per week. Other potentially useful strategies to mitigate faculty workload are assigning marks to students to take turns leading discussion forums and summarizing key points. Nevertheless, care should be taken when utilizing such strategies as this study indicates that students do value and anticipate the active participation of faculty in online discussions.

In addition to implementing strategies to strengthen the quality and timeliness of feedback to students, faculty members have control of the content and structure of courses. The respondents valued courses that are well organized, current, and appropriate to their learning needs. This is similar to what has been reported elsewhere [1]. 

Both flexibility and choice within courses and web discussions were also seen as important by these graduate nursing students. Our study did not make a clear distinction between flexibility and choice. The students in this study valued flexibility in deadlines for assignments and would welcome more flexibility in these deadlines. They would view positively being given choice in course content, readings, assignments, the membership of small work groups, and due dates for completing different sections of the course. They would also appreciate courses that do not have mandatory web participation for all weeks of the course. These preferences are all within the control of the faculty member who designs a web course and have not previously been detailed in the literature. Consideration can be given to strategies that increase the choices students have, while maintaining care to ensure that students are still able to master course objectives.

Of particular interest is the mean of 3.7 for the item “my ideal program would be completed without a thesis or major project,” which indicates many would prefer an all-course graduate program, with no thesis or practicum requirement. This finding is disturbing, as the outcome of graduate programs should be students who are able to identify, implement, evaluate, and disseminate projects and studies that advance nursing knowledge. In light of this finding, an important component of a graduate program in nursing may be to increase students' awareness of the goals of graduate education.

A number of students felt that they did not learn as much in online courses as in campus courses. The mean score in this sample of students, 3.71, was lower than the 4.44 reported by Mitchell et al. [9] in a small sample of dental hygiene students. This is an important finding, which should be explored further to investigate if it is based on reality or the students' perceptions, and if this finding is consistent for different graduate nursing programs. If students actually do not learn as much in online programs, then the credibility of graduates and the program will be questioned. Ultimately, faculty and students may not be attracted to such programs. If students do learn as much but do not perceive this is the case, they may feel and act as if they had an inferior education.

These MN students did not support the addition of an on-campus course requirement for distance students. Thus, while many do not feel they learn as much in online courses, they appreciate the flexibility of programs offered on line. The majority of these nurses were employed more than 40 hours a week and many lived outside of the city where the university was located. Therefore, they may not have been able to register for a graduate program if they had to attend on-campus classes.

Limitations of this study include that only students in one graduate program in nursing were surveyed, the number of respondents was small and the data were collected in 2005. With progress being made in distance education, the implications for further research and support for the strategies identified may also be limited. However, the aspects identified as being under the control of individual faculty members remain under their control. Administrators and faculty members may find the results useful in identifying issues and strategies to explore to improve graduate students' experiences as online learners.

## 5. Conclusion

This is one of the first studies to investigate online students' perceptions of ideal program and course characteristics, their preference for choice, and their perceptions of learning and to articulate those characteristics that can be controlled by the individual faculty member. It is important that graduate students learn as much in online courses as in on-campus courses and feel that they do. Faculty members and administrators need to consider what they can do to help with student learning and to increase student choices to optimize both learning and satisfaction with the student experience.

## Figures and Tables

**Table 1:** Demographic characteristics of respondents.

Characteristic	Category	Number*	%*
Sex	**Female**	**37**	**82.2%**
	Male	3	6.7%
	Unknown	5	11.1%

Age	<29	11	24.4%
	30–39	11	24.4%
	**40+**	**17**	**37.9%**
	Unknown	6	13.3%

Hours per week worked at job	0–16	4	8.9%
	17–30	6	13.3%
	**31–40**	**22**	**48.9%**
	40+	7	15.6%
	Unknown	6	13.3%

Hours per week spent on course(s) Discussion forum	**2–5**	**13**	**28.9%**
	6–9	9	20%
	10–20	9	20%
	Unknown	14	31.1%

*Number of respondents and % of all 45 respondents.

**Table:** (a)

Items related to *ideal program *	Mean score	SD
My ideal program would provide for a leave of absence status without financial penalty	4.62	.61
My ideal program would have technical support available within 24 hours	4.57	.50
My ideal program would be supported by a strong administrative infrastructure (e.g., effective procedures for answering questions, registration)	4.45	.59
My ideal program would allow me to take a number of courses per semester	3.73	1.05
My ideal program would be completed without a thesis or major project	3.67	1.13
My ideal program would allow me to take only one course per semester	2.98	1.25

**Table:** (b)

Theme/Stem	Items related to *courses* within an ideal program	Mean score	SD
Content and structure	Courses are well organized, with structured content	4.58	.54
	Course readings/textbooks reflect current knowledge in the discipline	4.58	.50
	Courses have seamless navigation	4.48	.51
	Course content is appropriate to my learning needs	4.47	.50
	Courses have a strong relationship of content to assignments	4.42	.50
	Course readings/resources are accessible on line instead of having to obtain a hard copy	4.02	1.1

Collaboration and feedback	Courses provide opportunities for me to receive feedback about my ideas from course instructors	4.56	.55
	Courses require collaboration among learners	4.25	.69
	Courses provide opportunities for me to receive feedback about my ideas from other course participants	4.13	.59
	Courses provide opportunities for me to respond to the ideas of other course participants	4.04	.64
	Courses encourage team effort and community building among learners	4.04	.64

Discussion forum	Courses have frequent instructor interaction in discussion forums	4.16	.67
	Courses have frequent discussion forums	3.47	.97

Instructors	Instructors respond to my emails within 24 hours	3.78	1.22

**Table 3:** Mean scores for items related to preferences for *choice/flexibility* and *learning* in courses.

Items for *choice/flexibility in courses *	Mean score	SD
Courses provide for choice in assignment topics	4.40	.50
I should be able to exercise some autonomy in terms of meeting my own learning needs through my coursework	4.15	.58
I would prefer that all courses provided choice in terms of how many discussion forums I was required to participate in	4.08	.96
I prefer some autonomy in terms of which weeks of the course I do more or less of the coursework.	4.05	.96
I would prefer choice in working on assignments individually or within a group	4.05	.88
Courses provide for flexibility in deadlines for individual work	4.02	.89
Courses do not have mandatory participation for all weeks of the course	3.98	1.08
Courses provide for flexibility in deadlines for group work	3.91	.87
Choice, in relation to course content issues, is desirable	3.65	.83
I prefer some autonomy in terms of when I work on different sections of the course	3.60	.96
Choice, in relation to readings and resources, is desirable	3.43	.93
I would prefer to choose my own partners/team members, rather than be assigned by the instructor	3.30	1.07

Items related to *learning *	Mean score	SD

I feel that I can apply my new knowledge to new and different situations in my professional life	4.13	.80
I feel that I have integrated the knowledge that I have learned and am able to relate it to my professional practice	4.03	.87
I feel that I have mastered the essential content of my online courses	3.90	.55
I feel that I am achieving success as an online learner, in terms of mastering the knowledge base of my program	3.85	.78
I am satisfied with my level of learning in most of my online courses to date	3.84	.90
I feel that my learning outcomes from this program can be favorably compared with graduates from other similar programs	3.71	.90
I feel that my learning outcomes from online courses are as favorable as if I had taken the course on campus	3.08	1.26
